# Parental Narratives of Bonding and Relational Experiences with Preterm Infants Born at 23 to 24 Weeks—A Qualitative Descriptive Study

**DOI:** 10.3390/children10050793

**Published:** 2023-04-28

**Authors:** Anniina Väliaho, Liisa Lehtonen, Anna Axelin, Riikka Korja

**Affiliations:** 1 Department of Psychology and Speech-Language Pathology, University of Turku, 20014 Turku, Finland; 2 Faculty of Medicine, University of Turku, 20014 Turku, Finland; 3 Department of Pediatrics and Adolescence Medicine, Turku University Hospital, The Wellbeing Services County of Southwest Finland (Varha), 20521 Turku, Finland; 4 Department of Nursing Science, University of Turku, 20014 Turku, Finland

**Keywords:** extremely preterm infant, parent–infant bonding, parental participation, qualitative study

## Abstract

(1) Background. The birth of an infant at 23 to 24 weeks poses a significant challenge to healthy parent–infant bonding because of the high risk of infant loss and the prolonged separation. The aim of this study was to retrospectively explore the narratives of parents with children born at 23–24 weeks about their bonding formation and relational experiences. (2) Methods. This was a qualitative descriptive study conducted with Finnish parents of children born at 23 or 24 weeks of gestation. Twenty-nine mothers and eight fathers were retrospectively interviewed using a semi-structured interview about the bonding process with their infant during the period in the neonatal intensive care unit (NICU) and their later parent–child relationship. Parents’ narratives were deductively analysed according to a framework previously developed for studying parental bonding. (3) Results. Our results showed that several parents described the bonding process as natural or even easy despite the traumatic start to parenthood. Support from NICU staff and providing opportunities for participation in infant care and parent–infant physical closeness were reported to enhance the bonding process. Our study identified earlier parenting experience as a new element supporting bonding. (4) Conclusions. According to our results, essential elements enhancing parent–infant bonding in NICU settings seem to be supporting parents psychologically, promoting parent–infant closeness, and encouraging parental participation in the infants’ care, even with the most immature infants.

## 1. Introduction

Extremely premature children born at 23 and 24 weeks of gestation are a small but growing population as the result of increasing survival due to an active approach to their intensive care. In the United States, approximately 4500 infants are born at 23–24 weeks of gestation per year and 60–65% of them receive active resuscitation. [1] In a comparison study between high-income countries, the survival rates at 24 gestational weeks varied from 35% to 84%, depending on the quality of care and the attitude toward active care of the most immature infants. [2] The birth of an infant born at 23 to 24 weeks of gestation inevitably generates fear in the parent about the infant’s survival, as these infants are treated in a neonatal intensive care unit (NICU) usually for several months. The environment is a highly medicalised with limited parent–infant proximity and often prolonged separation; these conditions pose a threat to the healthy formation of parenthood and parent–infant bonding.

Early parent–infant bonding—the formation of an emotional bond between parent and infant—has been acknowledged as a key factor in a healthy child, with important influences on infant brain development as well as the child’s psychological well-being and social development [3,4]. Furthermore, early parent–infant bonding also forms the basis for the long-term parent–child relationship [4]. While the classical attachment theory of John Bowlby [5] focuses on the infant’s perspective in attachment and emphasises the infant’s need for proximity with the mother as a basis for bonding, other theories such as Daniel Stern’s maternal representations theory [6] have highlighted the parental elements of bonding. Both mothers and fathers already begin their bonding processes during pregnancy by forming mental representations of their future child [7,8]. After the birth, the parental bonding behaviour includes elements that are typical to the bonding of mammals with new-born offspring, such as nursing, grooming, and touch [9,10]. Furthermore, the new parent’s preoccupation and anxiety about their infant’s well-being are not only typical but rather are essential parts of parental behaviour during the bonding period [11,12].

The quantitative study of Ruth Feldman et al. (1999) [12] clearly highlights the challenges of the bonding process following a preterm delivery. Feldman et al. interviewed three groups of mothers: mothers of full-term infants maintaining continuous proximity with the infant, mothers of healthy premature infants who were separated from the infant, and mothers of very low birthweight (VLBW) infants experiencing potential loss and prolonged separation. By classifying the interviews using a categorisation of the elements of bonding [13], Feldman et al. found that maternal attachment behaviours and representations declined linearly with the duration of mother–infant separation. In addition, maternal preoccupations, such as anxiety about the infant’s well-being, were significantly lower in mothers of VLBW infants. The authors concluded that the fear of losing the infant and the prolonged separation caused a distortion of the bonding process among the mothers of VLBW infants, which is in line with John Bowlby’s attachment theory [5,12].

Several qualitative studies have identified obstacles preventing healthy bonding in the NICU in mothers of premature infants: the medical environment, separation, lack of parental participation, and the fear of losing their infant [14]. Mothers often struggle with difficult emotions such as failure and guilt that further complicate the bonding process and maternal role formation [15,16,17]. Moreover, in cases where mothers are afraid that their premature child might die, they tend to distance themselves emotionally and do not let themselves become attached to the child [14,16,18]. NICU fathers, although less studied, have been reported to struggle with feelings of helplessness and being out of control [19], thus acting as barriers to participating in the infant’s care, and the lack of physical contact with the infant signal a significant risk for healthy bonding with their child.

To our knowledge, there are no previous studies which have specifically explored the bonding experiences of parents of infants born at 23–24 gestational weeks. This group of parents is of special interest regarding the bonding process as they face a long hospitalisation and related separation and they are in a high risk of losing their baby. Therefore, healthy bonding between parents and their baby is clearly challenged. Our present study aims to fill this gap using a qualitative approach. We studied how parents retrospectively described their bonding and relational experiences with their preterm infants born at 23 to 24 weeks during their stay in the NICU and in later stages of childhood.

## 2. Materials and Methods

### 2.1. Study Approach, Setting, and Participants

The study employed a qualitative description approach, which is a type of qualitative research that has as its goal a comprehensive summary of events in the everyday terms of those events [20]. It was conducted as a part of a larger research project, with the intention of exploring the quality of life and functioning of children born at 23–24 gestational weeks. The study was carried out in Finland, a high-income country with high-quality neonatal care. Ethical approval for the study was granted by the Ethical Committee of the Finnish South-West Hospital District in December 2016. The COREQ Checklist (The Consolidated Criteria for Reporting Qualitative Studies) was used as a reporting guideline [21].

All children of the participating families had received active neonatal care in the NICU at Turku University Hospital. The hospital has 4000 deliveries per year and the NICU (level III) has over 500 admissions per year. The NICU also places a strong emphasis on family-centred care. During the study period, the staff were trained using the Close Collaboration with Parents training to deliver care in partnership with parents during the years 2009 to 2012 [22]. As the Finnish family policy includes an extensive family leave system, with paid maternal and paternal leave [23], it was possible for the parents to stay with their infants during the hospital care without jeopardising their work.

Purposive sampling was used: all families (n = 39) with a surviving child or with children born at 22–24 gestational weeks in Turku University Hospital between 2001 and 2014 had been asked to participate in the study. The families were first contacted by an NICU nurse and if the parents expressed their interest, they received a call from the first author AV to obtain more information about the study. In seven families, the parents decided not to participate, either because of a busy life situation or lack of interest. One family could not be reached and one family did not have a fluent enough capacity of Finnish to participate. Of the remaining 30 families, parents were asked to participate in a semi-structured interview study concerning the everyday life of their child, the perceived effects of being born prematurely, and their parenting experiences during the neonatal care period. Both parents were given a chance to participate, but it was informed that mothers and fathers would be interviewed separately. In 25 families, parents were married or cohabiting; in five families, parents lived separately either as single or with a new partner. The premature child had been the parents’ firstborn for 13 families; in 17 families, there were one or several siblings. All participating parents signed an informed consent form.

### 2.2. Data Collection

The interviews were conducted in 2017 by the first author AV and supervised by senior researchers RK and LL. One pilot interview was conducted before the research interviews. The interviews took place either in the homes of the families or in a research facility provided by the university. The choice of the location was left to the parents. If they chose to be interviewed at home, it was ensured that the interview setting was as quiet and peaceful as possible. Twenty-nine mothers and eight fathers were interviewed. There were three twin mothers, who were encouraged to recall their experience separately with each child. The length of one interview was approximately 1–1.5 h. All interviews were videotaped and later transcribed verbatim.

The semi-structured interview developed for the research project is explained in detail in Väliaho et al. (2021) [24]. The interview started with an open question to give the parents a chance to freely describe the life of the child and the family, followed by questions on six themes with pre-determined topics (somatic health, functioning, learning and attention, emotional well-being, social relations, parenting experience). For this study, we analysed the answers to the sixth theme, which included questions about overall parenting experience, e.g., parental bonding and the NICU period, (see Table 1). These questions had been designed together with an experienced neonatologist (LL) and psychotherapist focused on early parent–child interaction (RK).

In addition, some parents answered the first open question of the interview (“*Please tell me about the life of your child, from the beginning until today*”) in a way that included experiences about both the hospital period and their child’s life after discharge. These were also included in the analysis.

### 2.3. Data Analysis

Our analysis method was thematic analysis, which is defined by Braun and Clarke [25] as a method offering an accessible and theoretically flexible approach to analysing qualitative data. We chose the theoretical (deductive) thematic analysis, in which the analysis was driven by a particular theoretical basis and thus it was a more explicitly analyst-driven approach than inductive thematic analysis [25]. This decision was based on the fact that parent–infant bonding is a well-studied area, and inductive analysis would not necessarily have provided relevant new theoretical information on parent–infant bonding. As a theoretical basis for our analysis, we used the elements of bonding categorised by Feldman et al. [12]: the YIPTA (The Yale Inventory of Parental Thoughts and Actions) [13]. Feldman et al. used the YIPTA categorisation to compare the bonding processes between mothers of preterm infants and mothers of full-term infants. While our study explored the bonding experiences parents of extremely preterm infants, our qualitative approach was notably different from Feldman et al. Therefore, the method YIPTA was assessed in detail for its appropriateness for this study, and some modifications to the categorisation were made as presented in detail in Table 2.

Before the main analysis, the interview data were read through several times to explore the global themes and impressions [25]. The data text was then systematically coded based on the categories described in Table 2. The coding of the data began by identifying features, or data extracts, in the text—words, sentences, or paragraphs—containing relevant information according to the theoretical categorisation that was used. These features were then labelled with codes and classified into the most appropriate category (AB, C, D, E, F, or Z). Within each category, several sub-categories were formed as a result of the coding process [25]; for example, in the category AB, a sub-category of mothers’ need for emotional distance. The process of coding and classifications was conducted by the first author AV and supervised by the other authors AA, RK, and LL. Throughout the process of coding and categorisation, the authors had mutual discussions for comparison, critical assessment, and validation.

## 3. Results

We present six categories (AB–F) of the elements of parent–infant bonding in extremely preterm infants, derived from our deductive analysis based on the categories used by Feldman et al. [12]. Additionally, we present the category Z that emerged from our analysis. Within each category, we describe smaller sub-categories formed in the coding process.

In each section, we start by describing content that arose in parents’ stories regarding the first weeks and months of their child’s life (the neonatal intensive care period and the time after discharge), followed by content regarding the time when the child was older.

### 3.1. AB: Thoughts, Worries, and Distress

Parents’ expressed feelings and thoughts of distress regarding their baby during the neonatal intensive care period. Their stories included descriptions about fears for their child’s safety, well-being, and survival, as well as grief about their baby having to endure such pain and discomfort. They mentioned traumatic memories related to surgeries or sudden deteriorations in their child’s condition. Parents described their feelings during those moments as “desperate”, “anxious”, or “worried about the future”. Some parents had vivid memories of specific aspects of a typical NICU environment, such as the sound of the ventilator, causing them anxiety. Parents experienced fear in cases where they had witnessed a bad or lethal outcome of another baby in the NICU.

“*The constant fear of death. And the worry. You are living with that fear 24/7, and you sleep holding your phone, in case you get a call from the NICU, and you are afraid of that call*.”

M12

Some mothers had felt a need to keep an emotional distance to their child in the beginning because of the uncertainty of the baby’s survival. They described that it was difficult for them to become attached to the baby, especially if they had not been able to hold their baby initially. Still, most recalled having had strong emotional feelings even in the beginning.

“*[The attachment] didn’t form at once, of course not, it couldn’t have, when it wasn’t possible to hold him … though I could touch him, he was my son, the emotional bond was strong even then*.”

M19

Another common experience was that parents could not recall details, which they explained by the neonatal intensive care period feeling “unreal” or “like being in a bubble”. As one mother expressed it “*I feel like I don’t know whether I was living [the NICU period] or not*” (M39). Fathers experienced “double worry” for their baby, as well as for their spouse who was recovering both physically from the birth and mentally from the realisation that their child needed intensive care.

When the preterm born child was older, parents’ thoughts, worries, and distress were related to the challenges and difficulties that prematurity had caused to the child and the whole family. Parents reported that the traumatic memories from the NICU period had influenced both family life and the parent–child relationship even years later. One mother described that for them as parents, normal, everyday-life challenges felt unnecessarily strong. Re-activation of the traumatic memories could happen when the child became even mildly ill or when seeing a hospital.

### 3.2. C: Distress Management

A great majority of mothers and fathers described that the support and encouragement from NICU staff had a significant role in reducing their stress and anxiety. Parents praised the staff’s professional knowledge and the quality of care their babies received, as well as the kindness and helpfulness of the staff. The parents felt they had been welcomed into the NICU; they could describe the NICU “like home”.

“*I am really grateful to the NICU staff. They were very capable in taking care of premature babies, but they also took care of us parents*.”

M4

Parents described that the encouragement that the staff offered to the parents, in their roles as caretakers of their babies, had played a vital role; without it, they would not have had the courage to take an active part in infant care. Mothers mentioned discussions with other NICU parents as distress-relieving. Fathers typically brought up the significance of work arrangements that had made it possible for them to spend more time in the NICU: they had been granted sick leave or they had been able to take a longer-than-planned vacation from their work.

Both mothers and fathers described their way of approaching life as a stress modifier. Some parents described having had, from the beginning, a strong confidence that their baby would make it. Through this belief they avoided thinking about worst-case scenarios regarding survival or prognosis. Some parents described personality traits helping them to cope, such as positivity, a sense of humour, the ability to take one day at a time, or being a laid-back person not prone to anxiety.

“*In general, I am a positive person. I will always believe in the good*.”

F25

When the preterm born child was older, some parents viewed their children’s journey as an example of “*per aspera ad astra*”, “through hardships to the stars”, as a distress management strategy. Parents expressed their thankfulness over their child having survived the rough start and presently doing so well. The parents whose children had developmental challenges tended to approach those as not being that significant from a broader perspective. This attitude supported the parents through challenging times even later in the life of their child.

### 3.3. D: Compulsive Checking

Compulsive checking did not emerge as a common theme in parents’ descriptions of the neonatal intensive care period in the participating parents. Some mothers did describe their discomfort when they had had to be away from the hospital, or having been afraid of the phone ringing when they were at home, as it might have been a call informing them of bad news.

“*The few times I was at home, taking a break from the NICU, felt really hard. I was pressurized to go by the hospital staff, they told me to go as I had an older son there … but it didn’t do me good. I felt I should be there with the baby, all the time*.”

M31

Parents also described having been anxious about the discharge, as they had been doubting whether they would be able to take care of the baby’s needs. Mothers, especially, emphasised their efforts to learn about the caretaking of their baby as well as possible.

Most of the content that could be classified into the compulsive checking category was found in parents’ stories regarding the first weeks and months after discharge. This was a time of anxiety and uncertainty for many parents. The parents described feeling the need to frequently check that their sleeping baby was breathing, while some mothers recalled even being scared to fall asleep themselves because of this.

“*Perhaps the most difficult time period was just after discharge from the NICU, at least for me. Because she still had times when her breathing was bad, and […] it was supposed to be our job to assess when it might be [more serious]*.”

F29

Parents’ desire to do “all they could” for the baby, including both caretaking and rehabilitation, was also classified into the category of compulsive checking. Moreover, although not a prominent theme in our sample, some parents described feeling overprotective of their child even long after infancy, such as worrying about leaving the child in the care of day care providers or even the child’s other parent.

“*I’m still anxious. Now I worry about her starting day care, although I realise that I’m overprotective*.”

M37

### 3.4. E: Affiliative Behaviour and Caretaking

Although the possibilities to express affiliative behaviour, such as touching and nursing, were limited when the babies were receiving intensive care, there were still elements of affiliative behaviours in parents’ stories. They described stroking the baby’s hand, singing a familiar song, reading children’s books, or saying “good night and see you tomorrow” to the baby each evening before leaving the NICU. Both mothers and fathers experienced that during the neonatal care period, the possibility to actively participate in and take responsibility for caretaking, such as changing diapers, bathing, and feeding the baby, had been relevant for the bonding process between them and their child.

“*Thinking back, I feel it was the most important thing, that I got to be with him and do some of those [caretaking] things and to hold him*.”

M13

Active participation and practical caretaking had increased confidence in parenting skills. Some mothers expressed that as their responsibility in caretaking increased, they did not want to let the nurses care for their baby anymore. Additionally, both mothers and fathers strongly emphasised the importance of physical closeness, especially kangaroo care, in the bonding process with their child.

“*Kangaroo care was really important. It made me feel at once that [my daughter] was my child*.”

F24

When the child was older, the parents still described elements of physical and/or emotional closeness in the relationship between them and the child. Some even felt a connection between kangaroo care in infancy and having lots of physical closeness later in the child’s life.

### 3.5. F: Parental Representations

Both mothers and fathers tended to attribute tenacity to their child as a very premature baby. Describing the neonatal intensive care period, parents used expressions such as the baby having been “*a fighter*”, “*a survivor*”, “*resilient*”, or “*perseverant*”. Furthermore, parents often considered that tenacity to be a quality that the baby had needed to overcome the rough start and the intensive care. Some babies that had developed exceptionally well during the intensive care period had been called a “*super-baby*” by the staff, and the parents had also taken to this name. Another representation that was presented by multiple parents was referring to the baby as “*the miracle child*”.

The opposite kind of representation was to describe the baby with expressions of fragility and smallness—“*the little weakling*”, “*little bird*”, “*poor thing*”. These representations came most often from the mothers and were not used as frequently as the attributes of resilience and perseverance. However, tenacity and fragility did not necessarily rule out one another, as in the mothers’ stories, the child could first be described as “a weakling” and a few sentences later as a “a fighter”.

Surprisingly, a common representation mothers and fathers shared was that they had experienced the baby “being theirs” from early on or at once having a strong bond. Many parents did not recall having difficulties in loving their baby.

*M24: Perhaps it pinned us together, or like … The bond with the child became especially tight because we went through stuff like that in the beginning with her. Somehow, for me it was more that way that it felt really easy to bond with my child, and like, to fall in love with the child at once*.

M24

However, as we have explained in detail in category AB, some mothers did describe being so afraid of losing their baby that they had trouble becoming attached. Nevertheless, even these mothers described that the bond between themselves and their baby had started to develop quite quickly after the most uncertain first days. Only one mother in the study expressed having had the feeling that the baby belonged to the hospital.

Parents tended to describe even older children as tenacious or resilient. Even the demanding characteristics of the child, such as stubbornness, could be seen through the lens of resiliency or perseverance. Furthermore, they emphasised the role of the NICU experience in that it had made their relationship with their child more “special”, often very close emotionally.

“*She’s a very positive, an amazingly tenacious girl.[…] And that’s what she’s always been. She’ll never give up trying*.”

M17

### 3.6. Z: Earlier Parental Experience

Some content in our data that could not be classified into the framework of Feldman et al. [12] was how the birth order of the child affected the formation of the parent–child relationship. Parents with older children tended to see that having had a child or children before the premature baby was beneficial for bonding. For mothers especially, but even for some fathers, a common theme was to compare the attachment process with the prematurely born child to the process they had experienced with their older child/children. Several mothers and one father concluded that bonding with the premature infant, even amidst all the uncertainty, had felt natural, just as it had felt with the older children.

“*I didn’t find [bonding with my son] difficult. I have wondered if that might be because he was my second child*.”

M13

In comparison, mothers, for whom the premature child had been their first, described their initial emotional confusion with added meaning because it had been such a new situation for them.

“*My first child, and a preemie. I didn’t know what I should do*.”

M30

“*Growing into motherhood with [my firstborn daughter] hasn’t been an easy task*.”

M27

An interesting variation in this category were families with twins. Three twin mothers described that during the neonatal intensive care period, they had often felt insufficient as parents, as there had been not only one but two babies. Often the progress of the twins had not been similar, and one of them could be doing better while the other was struggling, thus adding to the parents’ confusion. One twin mother described a situation where one of her babies was already discharged from the hospital, but they did not want to separate the children from one another, so they brought the discharged baby to spend nights at the hospital to be with her brother. Another family had had to make a difficult decision to let their grandmother care for the discharged twin, while the parents remained in the hospital with the other baby. An even more demanding situation that some twin mothers had faced was the loss of one twin while the other had survived. Naturally, this led to a severe emotional struggle to simultaneously grieve for the baby they had lost and try to attach to the other baby who was in an unstable condition.

Fathers, for their part, pondered about the role of siblings more from the perspective of parenting in general. They reported that parenting any of their children had evoked similar emotions for them, including feelings of joy and hopefulness and sometimes worry and stress.

## 4. Discussion

Our study focused on parents’ narratives on the bonding process and later relational processes with their children, in a group of infants born at 23 and 24 weeks of gestation. Parental experiences were deductively analysed with the YIPTA framework developed by Feldman et al. [12]. The parents’ experiences mostly corresponded with the YIPTA framework and similar elements of bonding were identified as important as in the study by Feldman et al. In our study, several parents described the bonding process with their baby as natural or even easy despite the traumatic start to parenthood, whereas previous evidence suggests that in VLBW mothers, the fear of losing the infant and the prolonged separation can cause a distortion in the bonding process [12]. The parents emphasised that their bonding process was enhanced by support from the NICU staff, the opportunities for participation in infant care, and parent–infant physical closeness. These elements in care were also of help to parents when coping with parental feelings of distress. In addition, our study identified the earlier parenting experience as a new element supporting bonding.

Our study included both mothers and fathers sharing their experiences, but mothers (n = 29) outnumbered fathers (n = 8). There were no robust differences between mothers’ and fathers’ narratives. Still, as presented in the results section, some sub-categories were emphasised more clearly in either mothers’ or fathers’ stories. For example, in the category of distress management, describing flexibility in work arrangements to provide time in the NICU was a more prominent sub-category in fathers’ narratives, while emphasising the importance of fellow NICU parents was more common for mothers.

The parents tended to express their representations of the child with a positive emphasis, both regarding infants and older children. They recognised the developmental or behavioural challenges of their children but regarded them as “not relevant or important in the bigger picture”. Earlier research in VLBW children has focused more on the challenges of the child and of the families and less on the positive aspects of the child. However, positive perspectives from the NICU experiences of parents have also been reported [26,27]. It is possible that the qualitative interview with the open questions provided space for parents to share also their positive experiences as well as challenges. The positive representations may also be a result of survival after a critical neonatal period and related worry and fear. Overprotectiveness and idealised representations, found in earlier studies with parents of VLBW children [28,29], may also be manifestations of a distorted attachment.

Even if parents saw their children from a positive perspective, some parents reported difficulties in their bonding process, such as fear of becoming attached to their child. Generally, parents expressed a considerable number of traumatic memories, worries, and emotions concerning the NICU period. A common theme regarding later relational experiences was the re-activisation of traumatic elements from the NICU period, which is in line with the literature about later PTSD (post-traumatic stress disorder) symptoms in parents of premature children. [30]

Interestingly, our data did not provide support for parents’ compulsive checking during the NICU stay and most of these experiences emerged after discharge to home. The explanation might be that the parents trusted the care provided by the staff during the NICU period and the full responsibility of the care at home contributed to the occurrence of the behaviour of compulsive checking. In the study of Feldman et al. [12], compulsive checking occurred less frequently among the mothers of VLBW infants compared to full-term and LBW infants.

The parents in our study emphasised the support they had experienced provided by the NICU staff and sometimes even other NICU parents. The importance of the support by neonatal unit staff has been stated in several qualitative studies [14]. According to the mothering theory of psychologist Daniel Stern [31], new mothers do not only need advice but also a positive psychological holding condition that lets them to feel free to explore and find their natural maternal behaviour. In our study, the hospital staff and peer parents might have played a role in this positive psychological holding of the parents. The NICU in which our study was conducted placed a strong emphasis on family-centred care (FCC) [32], which might have provided the parents more support and thus facilitated the bonding process. In addition, the hospital staff had undertaken the Close Collaboration with Parents education which had provided them with the skills to support parents and parenting [22]. Our study is in line with previous studies showing that practices of FCC can influence parenting by supporting parental mental health [33] and bonding [34]. Moreover, it is worth noting that our study was conducted in a high-income country with a generally high parental education level and a generous family leave system that provided parents opportunities to be present in the NICU for prolonged time periods.

Other elements that enhanced bonding were the opportunities for participation in infant care and parent–infant physical closeness. Earlier literature shows that an educational–behavioural intervention program to enhance mothers’ participation improved mother–infant interactions scores [35]. Recent literature has reported very positive and meaningful parental experiences related to immediate physical parent–infant closeness among parents of very preterm infants [36,37,38,39]. In addition, immediate skin-to-skin contact was associated with less impaired bonding in a randomised controlled trial including infants born between 25 and 32 weeks of gestation [40].

Our study contributed earlier parenting experience as a new element that might affect bonding. The parents participating in our study reported that the earlier positive bonding experiences were of help, whereas more difficult bonding processes were found in the stories of first-time parents. A more detailed perspective of earlier parental experience might be a relevant question in future studies of parent–infant bonding.

### 4.1. Strengths and Limitations

One of the strengths of our study was including stories from both mothers and fathers, as the fathers’ perspective is often missing from studies of NICU parents. Another strength in this study was the well-defined target group, as we focused on the families of the most immature preterm infants requiring a long hospitalisation with a high risk of death and the long-term consequences of prematurity. Furthermore, the majority of parents approached agreed to participate in the study. The parents’ stories were rich and included a considerable amount of personal and intimate experiences, signalling a good confidential relationship between the researcher and parents, as well as credible results.

Our study also has limitations. As the parental bonding process already begins during pregnancy, one limitation with the method is not having included questions about pregnancy in the interview. Another limitation to be considered is the framework used for the analysis; the YIPTA had originally been used to interview parents soon after the birth [12], while the time lag varied in our study from the NICU experiences to the interview. Due to this, our interview structure differed from the original interview in the YIPTA framework. The retrospective approach of our study also limits the trustworthiness. However, this study focuses on parents’ representations of their bonding process, which are dynamic constructs and meaningful for a child’s well-being and development, regardless of their accuracy. It is natural that parental representations of the bonding process had developed and been influenced by later life events. Parents recalled memories about the bonding process retrospectively, some of them even years after the actual NICU experience. Still, the parental interviews contained many detailed and vivid descriptions concerning the bonding during and related to the NICU experience, as illustrated in the citations in the results section. In addition, due to the small annual numbers of infants born at 23 to 24 gestational weeks, the retrospective approach was the most appropriate way for our study to reach a sufficient number of families to gain saturation in the categories.

### 4.2. Future Studies and Clinical Implications

In future studies regarding bonding and relational experiences of parents of very premature infants, one important theme might be the developmental pattern between bonding experience and the later parent–child relationship. Another theme for future studies is the role of pregnancy-related factors; for example, whether prematurity is anticipated or unexpected and how this affects the parental bonding experience. Our study has clinical implications in the development of neonatal care practice and interventions that aim to support the bonding process between premature infants and their parents.

## 5. Conclusions

In our study about parental experiences of bonding with their infants born at 23–24 weeks of gestation, parents retrospectively described both positive and negative experiences. Many parents described the bonding process as natural or even easy while still recalling the traumatic start to parenting. The supportive elements reported by parents to enhance the bonding process were psychological support from NICU staff, promoting parent–infant closeness, and encouraging parental participation in the infant’s care.

## Figures and Tables

**Table 1:** Interview questions analysed for this study.

*Your child was born extremely premature, and (s)he needed a long neonatal intensive care period at the hospital. Today, how do you think being born prematurely has affected the life of your child?* *I will now ask some questions about the neonatal intensive care period. Could you describe the neonatal intensive care period? How did you feel during that time?* *Are there any particular events that you still remember from the NICU?* *How would you describe the development of the bond between you and your child in the beginning? What was helpful to you in bonding with your child?* *At what phase did you start to have confidence that your child would survive?* *Could you please describe the memories you have about the first days and weeks of having your child at home?* *Now, I am asking you to think about the current situation again. Could you please describe the way you feel about being the mother/father of your child?* *What brings joy to you about parenting your child?* *What are the challenges or stressors you find in parenting your child?*

**Table 2:** Modifications from the YIPTA.

*Name of Category in the YIPTA*	*Content of Category in the YIPTA (Feldman et al., 1999 [12])*	*Name of Category in Our Study*	*Content of Category in Our Study*
*A: Frequency of Thoughts and Worries*	typical thoughts and worries mothers experience in the immediate post-partum period	Combined category AB: *Thoughts, worries, and distress*	- thoughts, worries, emotions, and frightening situations parents described during the neonatal intensive care period and after discharge - parental worries about an older child and the effects that prematurity had had on the child
*B: Distress Caused by Thoughts and Worries* ^1^	level of distress caused by thoughts and worries in the immediate post-partum period		
*C: Distress Management*	strategies for managing the distress caused by thoughts and worries	*Distress Management*	strategies parents had to deal with their distress during the neonatal care period, after discharge, and later in the child’s life
*D: Compulsive Checking*	- the frequency of the mother checking her infant - maternal anxiety in cases when checking was prevented	*Compulsive checking*	- situations of frequently checking the infant during the neonatal care period and after discharge - checking and overprotective parental behaviour later in the child’s life - parental anxiety in cases when checking was prevented
*E: Affiliative Behaviour*	- the repetitive behaviours typical of maternal caretaking and interactions with new-born infants in mammals that appear to promote the selectivity of the mother–infant bond	*Affiliate Behaviour and Caretaking*	- affiliative and caretaking behaviours that were possible in the NICU environment, such as changing nappies, feeding, and kangaroo care - affiliative behaviour, caretaking, and closeness with an older child
*F: Attachment Representations*	- mother’s emerging internal model of her child - the mental indicators of bonding that point to the uniqueness of the bond in the mother’s mental life	*Parental representations*	- descriptions, mental images, or explanations that parents used to describe their child and his or her uniqueness or personality
*H: Frequency of Caretaking behaviour*	- mothers were asked to estimate the cumulative time per day devoted to various caretaking activities	-	[not applicable retrospectively]
-	-	*Z: Earlier parental experience*	- parental narratives about having had a child or children before the premature child that affected the bonding experience—content that could not be classified into the categories of the YIPTA

^1^ Category B in the YIPTA assessed levels of distress, which we had not explored quantitatively in our study; however, in our interviews the parents described their feelings of distress qualitatively. Thus, we combined categories A and B to one category as AB.

## Data Availability

The data are not publicly available due to individual privacy and the sensitive nature of the interviews.
